# Management and treatment of severe immune-related hepatotoxicity based on clinical and pathological characteristics

**DOI:** 10.1007/s12072-024-10688-0

**Published:** 2024-07-02

**Authors:** Nan Zhang, Zhaohui Li, Yutao Liu, Xiaohua Shi, Di Shi, Yue Li, Xiaoyan Si, Ziyu Xun, Jing Shao, Haitao Zhao, Hanping Wang

**Affiliations:** 1 Department of Liver Surgery, Peking Union Medical College Hospital, Chinese Academy of Medical Sciences & Peking Union Medical College, No.1 Shuaifuyuan, Beijing, 100730 China; 2 Division of Pulmonary and Critical Care Medicine, State Key Laboratory of Complex Severe and Rare Diseases, Peking Union Medical College Hospital, Chinese Academy of Medical Sciences & Peking Union Medical College, No. 1 Shuaifuyuan, Beijing, 100730 China; 3 Department of Medical Oncology, National Cancer Center/National Clinical Research Center for Cancer/Cancer Hospital, Chinese Academy of Medical Sciences and Peking Union Medical College, Beijing, 100021 China; 4 Department of Pathology, Peking Union Medical College Hospital, Chinese Academy of Medical Sciences & Peking Union Medical College, Beijing, China; 5 Department of Emergency Medicine, Peking Union Medical College Hospital, Chinese Academy of Medical Sciences & Peking Union Medical College, Beijing, China; 6 Department of Digestive Medicine, Peking Union Medical College Hospital, Chinese Academy of Medical Sciences & Peking Union Medical College, Beijing, China

**Keywords:** Immune-related adverse event, Immune-related hepatitis, Immune checkpoint inhibitor-mediated hepatotoxicity, Drug-induced liver injury, Immunotherapy, Immune checkpoint inhibitor, Programmed death-1, Programmed death ligand 1, Cytotoxic T lymphocyte-associated antigen-4, Oncology practice

## Abstract

**Background:**

The management of severe immune-related hepatotoxicity (irH) needs to be further optimized. This study aims to analyze the clinical characteristics of severe irH; improve the therapeutic strategy, especially salvage treatment in steroid-refractory irH; and determine the safety of immune checkpoint inhibitor (ICPi)-rechallenge.

**Methods:**

This multicenter retrospective study included patients who developed severe irH and those without irH after immunotherapy between May 2019 and June 2023. Propensity score matching was used to match these two cohorts with similar baseline characteristics.

**Results:**

Among 5,326 patients receiving ICPis, 51 patients developed severe irH. irH occurred after a median duration of 36 days and a median of two doses after the first ICPi administration. Patients receiving PD-L1 inhibitors faced a lower risk of developing severe irH. A higher dose of glucocorticoids (GCS) was administered to grade 4 irH than grade 3 irH. For steroid-sensitive patients, grade 4 irH individuals received a higher dosage of GCS than those with grade 3 irH, with no difference in time to resolution. Meanwhile, a significantly higher dose of GCS plus immunosuppression was needed in the steroid-refractory group. Liver biopsy of the steroid-refractory patients exhibited heterogeneous histological features. Twelve patients were retreated with ICPi. No irH reoccurred after a median follow-up of 9.3 months.

**Conclusion:**

irH requires multidimensional evaluation. PD-L1 inhibitors correlated with a lower risk of severe irH. Grade 4 irH demands a higher dose of GCS than recommended. Pathology may guide the salvage treatment for steroid-refractory irH. ICPi rechallenge in severe irH is feasible and safe.

**Supplementary Information:**

The online version contains supplementary material available at 10.1007/s12072-024-10688-0.

## Introduction

In recent years, immunotherapy has shown significant progress in improving the survival of patients with advanced carcinoma [1, 2]. Immune checkpoint inhibitors (ICPis) enhance antitumor immune responses by blocking endogenous immune-downregulated factors such as cytotoxic T lymphocyte antigen-4 (CTLA-4), programmed death protein-1 (PD-1), and programmed death ligand 1 (PD-L1). By increasing the activity of the immune system, ICPis can cause inflammatory side effects, which are often termed immune-related adverse events (irAEs). Although irAEs can affect any organ, the gastrointestinal tract, endocrine glands, skin, lungs, and liver are the most commonly affected sites [3].

The incidence of immune-related hepatotoxicity (irH) ranges from 2 to 10% in single-agent immunotherapy [4–6], and the incidence of high-grade irH (grade 3–5) reaches 1–2% [7]. Although death due to irH is less frequent than immune-related colitis, pneumonitis, and myocarditis, irH still accounts for 8–11% of ICPi-related deaths in the same cohort according to a systematic review and meta-analysis [8]. At present, no strong randomized evidence exists to guide the management of severe irH, and retrospective studies are still rare. According to the treatment guidelines for severe irH, glucocorticoids (GCS) and mycophenolate mofetil (MMF) are the treatment cornerstones for this irAE [9, 10]. However, this regimen showed poor therapeutic efficacy in steroid-refractory irH, especially in immune-related cholangitis [11, 12], and the alternative options are still unclear. Therefore, it is necessary to explore the clinical characteristics of severe irH and improve therapeutic strategies, especially salvage therapy for steroid-refractory irH. This study aimed to analyze the process management of both severe irH and steroid-refractory irH and to test the safety of ICPi rechallenge.

## Patients and methods

### Study design and patients

**Fig. 1:**
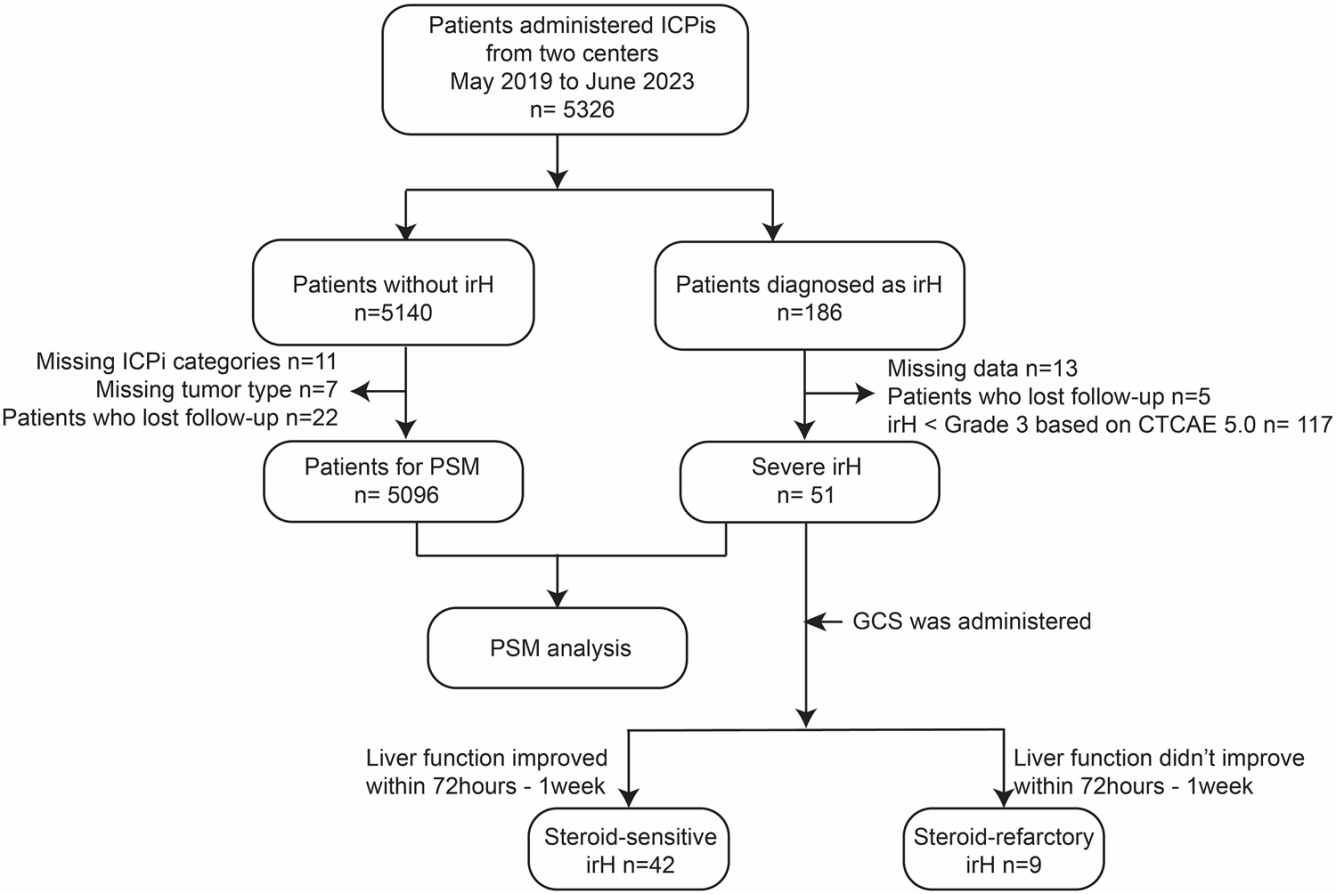
Flowchart of the enrolled procedure**.** *CTCAE V5.0* Common Terminology Criteria for Adverse Events Version 5.0, *GCS* glucocorticoids, *irH* immune-related hepatotoxicity,* ICPi* immune checkpoint inhibitor, *PSM* propensity score matching

This was a multicenter retrospective study involving Peking Union Medical College Hospital and Cancer Hospital Chinese Academy of Medical Sciences. Patients who developed severe irH and those without irH after immunotherapy were enrolled (Fig. 1). Age and sex were not restricted.

All cases were discussed at a multidisciplinary board comprising at least one radiologist, one liver surgeon, one oncologist, and one hepatologist. The causal relationship between immunotherapy and irH was assessed using the Roussel Uclaf Causality Assessment Method (RUCAM) [13]. A series of studies were performed to exclude other potential causes, including viral hepatitis serology, autoimmune serology, viral load (cytomegalovirus (CMV) and Epstein–Barr virus (EBV)), and liver imaging (abdominal ultrasound or computerized tomography) to exclude biliary obstruction. Patients with genetic and metabolic liver disorders were also excluded. The severity of irH was determined according to the Common Terminology Criteria for Adverse Events Version 5.0 (CTCAE V5.0) [14]. All patients were required to discontinue ICPi and were routinely monitored daily or every other day for laboratory parameters. According to the drug-induced liver injury (DILI) working group criteria [13], the biochemical classification of liver injury was defined as a hepatocellular, mixed, or cholestatic pattern based on liver function. According to the American Society of Clinical Oncology (ASCO) guidelines [9], liver biopsy was performed when it was defined as steroid refractory in the available patients. The probability of rechallenge with ICPis was evaluated by two senior oncologists. For those who retreated with immunotherapy, periodic visits were required and blood tests were performed before each ICPi infusion.

### Methods

For the entire cohort, demographics and clinical variables were recorded. Specifically, the ICPi cycle, time to onset (TTO, defined as the duration between the first ICPi and irH), clinical symptoms, and other key laboratory parameters (Supplementary Methods) were also recorded in the severe irH group. As for irH treatment, the GCS dose, time to resolution (TTR, defined as the time between the administration of the GCS and the normalization of liver function), immunosuppressants, and complications were recorded. The demographic and clinical variables, retreatment regimen, and accompanying irAEs were also collected in the rechallenge group.

Liver biopsies were reviewed by at least two pathologists focusing on the following features: interface hepatitis (0–4), focal necrosis, apoptosis, focal inflammation (0–4), confluent necrosis (0–6), and portal inflammation (0–4) according to Ishak’s hepatitis activity index. The bile duct situation (bile duct reaction, bile duct injury, and bile duct loss) and immunocytochemistry (CD3, CD4, CD8, CD34, CK7, CK19, and CK AE1/AE3) were also observed.

### Statistical analysis

Given the differences in the baseline characteristics between eligible participants in the severe irH group and non-irH group (Table 1), propensity score matching was used to identify a cohort of patients with similar baseline characteristics (Supplementary Methods).

**Table 1:** Baseline characteristics of the immunotherapy cohort before and after propensity score matching

Variables	Before matching		*p* value	After matching		*p* value
	Patients with severe irH (*N* = 51)	Patients without irH (*N* = 5096)		Patients with severe irH (*N* = 51)	Patients without irH (*N* = 51)	
Age, years^a^						
< 50	8 (15.7)	693 (13.6)	0.665	8 (15.7)	11 (21.6)	0.375
50–59	8 (15.7)	1402 (27.5)	0.06	8 (15.7)	7 (13.7)	1
60–69	26 (51.0)	1962 (38.5)	0.069	26 (51.0)	27 (52.9)	1
≥ 70	9 (17.6)	1039 (20.4)	0.629	9 (17.6)	6 (11.8)	0.25
Male, *n* (%)^a^	37 (72.6)	3722 (73.0)	0.938	37 (72.5)	37 (72.5)	1
Tumor type^a^						
Hepatobiliary carcinoma, *n* (%)	8 (15.7)	1535 (30.1)	0.025	8 (15.7)	8 (15.7)	1
Lung cancer, *n* (%)	20 (39.2)	2952 (57.9)	0.007	20 (39.2)	19 (37.3)	1
Other, *n* (%)	23 (45.1)	609 (12.0)	< 0.001	23 (45.1)	24 (47.1)	1
Stage IIIB–IV, *n* (%)^a^	46 (90.2)	4504 (88.4)	0.687	46 (90.1)	50 (98.0)	0.125
Diabetes, *n* (%)^a^	9 (17.6)	286 (5.6)	< 0.001	9 (17.6)	9 (17.6)	1
Hypertension, *n* (%)^a^	11 (21.6)	565 (11.1)	0.018	11 (21.6)	10 (19.6)	1
Prior myocardial infarction, *n* (%)^a^	2 (3.9)	29 (0.6)	0.037	2 (3.9)	0	0.5
Coronary artery disease, *n* (%)^a^	6 (11.8)	246 (4.8)	0.05	6 (11.8)	6 (11.8)	1
Heart failure, *n* (%)^a^	0	51 (1.0)	1	0	0	
Cerebrovascular disease, *n* (%)^a^	5 (9.8)	81 (1.6)	0.001	5 (9.8)	4 (7.8)	1
Hepatitis virus infection						
HBV infection, *n* (%)	12 (23.5)	645 (12.7)	0.021	12 (23.5)	5 (9.8)	0.065
HCV infection, *n* (%)	2 (3.9)	28 (0.5)	0.035	2 (3.9)	1 (2.0)	1
Liver cirrhosis, *n* (%)	1 (2.0)	120 (2.4)	0.853	1 (2.0)	3 (5.9)	0.625
Fatty liver disease, *n* (%)	2 (3.9)	32 (0.6)	0.044	2 (3.9)	0	0.5
Primary biliary cirrhosis, *n* (%)	0	3 (0.1)	1	0	0	
Autoimmune diseases, *n* (%)^b^	3 (5.9)	36 (0.7)	0.007	3 (5.9)	0	0.25
Alcohol intake, *n* (%)	12 (23.5)	308 (6.0)	< 0.001	12 (23.5)	5 (9.8)	0.092
Liver metastasis, *n* (%)	9 (17.7)	579 (11.4)	0.16	9 (17.6)	10 (19.6)	1
ICPi categories						
Anti-PD-1, *n* (%)	46 (90.2)	4129 (81.0)	0.096	46 (90.2)	39 (76.5)	0.092
Anti-PD-L1, *n* (%)	3 (5.9)	1304 (25.6)	0.001	3 (5.9)	16 (31.4)	0.004
Anti-CTLA-4 + anti-PD-1/PD-L1, *n* (%)	2 (3.9)	28 (0.5)	0.035	2 (3.9)	1 (2.0)	1
Combination therapy						
Target therapy alone, *n* (%)	9 (17.6)	1065 (21.0)	0.57	9 (17.6)	9 (17.6)	1
Chemotherapy alone, *n* (%)	21 (41.2)	2242 (44.0)	0.687	21 (41.2)	25 (49.0)	0.541
Target plus chemotherapy, *n* (%)	5 (9.8)	1041 (20.4)	0.061	5 (9.8)	10 (19.6)	< 0.001

*CTLA-4* cytotoxic T lymphocyte antigen-4, *HBV* hepatitis B virus, *HCV* hepatitis C virus, *ICPi* immune checkpoint inhibitor, *irH* immune-related hepatotoxicity, *PD-1* programmed death protein-1, *PD-L1* programmed death ligand 1

^a^Matching variables

^b^In this entry, we excluded autoimmune hepatitis, primary sclerosing cholangitis, and primary biliary cirrhosis. Nobody in the entire cohort was diagnosed with autoimmune hepatitis and primary sclerosing cholangitis

Normally distributed quantitative variables were compared using Student’s *t* test and expressed as mean ± standard deviation. Variables with a non-normal distribution were analyzed using the Wilcoxon rank-sum test and are expressed as median and interquartile range (IQR). Categorical variables were compared using the Fisher’s exact test and were expressed as frequencies and percentages. In the matched cohort, paired comparisons were performed using McNemar’s test for binary variables. The results were considered statistically significant when the *p* value was less than 0.05. All statistical analyses were performed using IBM SPSS, version 26.0 (SPSS, Inc., Armonk, NY).

## Results

### General characteristics and risk factors associated with severe irH

Among the 5,326 patients receiving ICPis, 186 patients (3.49%) were diagnosed with irH, and 51 (0.96%) were diagnosed with severe irH. Table 1 delineates the baseline characteristics of the severe irH group and non-irH group, both pre- and post-propensity score matching. Subsequent conditional logistic regression analysis of other unmatched factors (Supplementary Table S1) revealed that patients undergoing PD-L1 inhibitor therapy exhibited a reduced risk of developing severe irH compared to those receiving other ICPis (*p* = 0.0343; odds ratio: 0.230, 95% confidence interval 0.05899–0.8969).

The detailed baseline characteristics of the severe irH group are documented in Table 2. Twenty-one (41.2%) patients experienced grade 3 irH, and 30 (58.8%) experienced grade 4 irH. The median age was 65 years (IQR, 57–68), and 37 patients (72.5%) were male. Twelve patients (23.5%) had HBV infection and 11 (21.6%) had a history of alcohol consumption. The vast majority of the primary tumors were lung cancer (20, 39.2%) and hepatobiliary carcinoma (8, 15.7%). Most of the patients were at stage IIIb–IV (46, 90.2%). Thirteen (25.5%) patients presented with liver lesions, of which nine had liver metastasis. The main ICPi administered were PD-1 inhibitors (46, 90.2%), although three patients (5.9%) received PD-L1 inhibitors and two (3.9%) received a combined anti-CTLA plus anti-PD-1 regimen. Fourteen (27.5%) patients received targeted therapy, and 26 (51%) received chemotherapy. The details about the primary tumor type and its therapeutic regimen are listed in Supplementary Table S2. There was no significant difference between patients with grade 3 and 4 irH in terms of general characteristics.

**Table 2:** The general characteristics of the patients with severe irH

Variables	Overall (*n* = 51)	Grade 3 (*n* = 21)	Grade 4 (*n* = 30)	*p* value
Age, years (median, IQR)	65 (57–68)	64(56–68)	66(59–69)	0.43
Sex, *n* (%)				
Male,* n* (%)	37 (72.5)	15 (71.4)	22 (73.3)	1
Female, *n* (%)	14 (27.5)	6 (28.6)	8 (26.7)	
Hepatitis virus infection, *n* (%)	13 (25.5)	7 (33.3)	6 (20.0)	0.338
HBV infection, * n* (%)	12 (23.5)	7 (33.3)	5 (16.7)	0.196
HCV infection, * n* (%)	2 (3.9)	1 (4.8)	1 (3.3)	1
Diabetes, *n* (%)	9 (17.6)	5 (23.8)	4 (13.3)	0.334
Hypertension, *n* (%)	11 (21.6)	7 (33.3)	4 (13.3)	0.173
Prior myocardial infarction, * n* (%)	2 (3.9)	1 (4.8)	1 (3.3)	1
Coronary artery disease, * n* (%)	6 (11.8)	1 (4.8)	5 (16.7)	0.391
Cerebrovascular disease, * n* (%)	5 (9.8)	2 (9.5)	3 (10)	1
Liver cirrhosis	1 (2.0)	0	1 (3.3)	1
Fatty liver disease	2 (3.9)	1 (4.8)	1 (3.3)	1
Autoimmune diseases	3 (5.9)	1 (4.8)	2 (6.7)	1
Alcohol intake, * n* (%)	11 (21.6)	7 (33.3)	4 (13.8)	0.166
Tumor type, * n* (%)				0.109
Lung cancer	20 (39.2)	5 (23.8)	15 (50)	
Hepatobiliary carcinoma	8 (15.7)	3 (14.3)	5 (16.7)	
Other	23 (45.1)	13 (61.9)	10 (33.3)	
Liver metastasis, * n* (%)	9 (17.6)	2 (9.5)	7 (23.3)	0.28
TNM stage, * n* (%)				1
IIIA	5 (9.8)	2 (9.5)	3 (10)	
IIIB-IV	46 (90.2)	19 (90.5)	27 (90)	
ICPi categories, * n* (%)				
Anti-PD-1	46 (90.2)	18 (85.7)	28 (93.3)	0.222
Anti-PD-L1	3 (5.9)	1 (4.8)	2 (6.7)	
Anti-CTLA-4 + anti-PD-1	2 (3.9)	2 (9.5)	0	
Combination therapy, * n* (%)				0.96
Target therapy alone	9 (17.6)	3 (14.3)	6 (20)	
Chemotherapy alone	21 (41.2)	9 (42.9)	12 (40)	
Target plus chemotherapy	5 (9.8)	2 (9.5)	3 (10)	
RUCAM score, *n* (%)				
Highly probable	34 (66.7)			
Probable	17 (33.3)			
TTO, days (median, IQR)	36 (21–85)	31 (19–72)	42 (23–91)	0.18
Number of ICPi cycle, median (IQR)	2 (1–4)	2 (1–3)	2 (1–4)	0.59
Number of the patients with symptoms, *n* (%)	34 (66.7)	7 (33.3)	27 (90)	< 0.001
Peak levels of laboratory tests, median (IQR)				
ALT (U/L)		351 (228–506)	828 (265–2099)	
AST (U/L)		291 (173–583)	561 (205–1347)	
GGT (U/L)		403 (135–688)	671 (185–1013)	
ALP (U/L)		270 (148–544)	251 (161–695)	
Tbil (mg/dL)		20 (8–55)	136 (53–234)	
Dbil (mg/dL)		15 (4–39)	105 (29–184)	
ANA titer ≥ 80 (*n*, %)	11(21.6)	2 (9.5)	9 (30)	0.098
Biochemical classification of liver injury, *n* (%)				0.373
Hepatocellular	25 (49.0)	10 (47.6)	15 (50)	
Mixed	8 (15.7)	5 (23.8)	3 (10)	
Cholestatic	18 (35.3)	6 (28.6)	12 (40)	
Rechallenge, *n* (%)	12 (23.5)	7 (33.3)	5 (16.7)	0.248

*ALT* alanine aminotransferase, *AST* aspartate aminotransferase, *GGT* gamma-glutamyl transferase, *ALP* alkaline phosphatase, *Tbil* total bilirubin, *Dbil* direct bilirubin, *ANA* anti-nuclear antibody, *HBV* hepatitis B virus, *HCV* hepatitis C virus, *ICPi* Immune checkpoint inhibitor, *RUCAM* Roussel Uclaf Causality Assessment Method, *DILI* drug-induced liver injury, *irH* immune-related hepatotoxicity, *TTO* time to onset, *IQR* interquartile range

According to the RUCAM assessment method, a causal relationship between immunotherapy and irH was highly probable in 34 (66.7%) patients and probable in 17(33.3%). irH occurred after a median duration of 36 (TTO; IQR, 21–85) days and a median of two (IQR, 1– 4) doses. The most common symptoms were jaundice (24, 47.1%), anorexia (18, 35.3%), fever (15, 29.4%), and vomiting (10, 19.7%). Most patients with grade 3 irH were diagnosed in routine examinations before each immunotherapy cycle, with only seven individuals (33.3%) experiencing symptoms compatible with irH. In contrast, in patients with grade 4 irH, most of them (27, 90%) experienced obvious systemic symptoms. Based on the biochemical classification of liver injury, the proportions of hepatocellular, cholestatic, and mixed patterns in grade 3 irH were 47.6%, 23.8%, and 28.6%, respectively. In grade 4 irH, these proportions were 50%, 10%, and 40%. Most of the patients showed a negative result of autoantibodies tests. However, 11 patients presented with positive ANA at low titers (1:80, *n* = 10; 1:100, *n* = 1). EBV-DNA and CMV-DNA tests were negative in all patients.

### Treatment and outcome

**Fig. 2:**
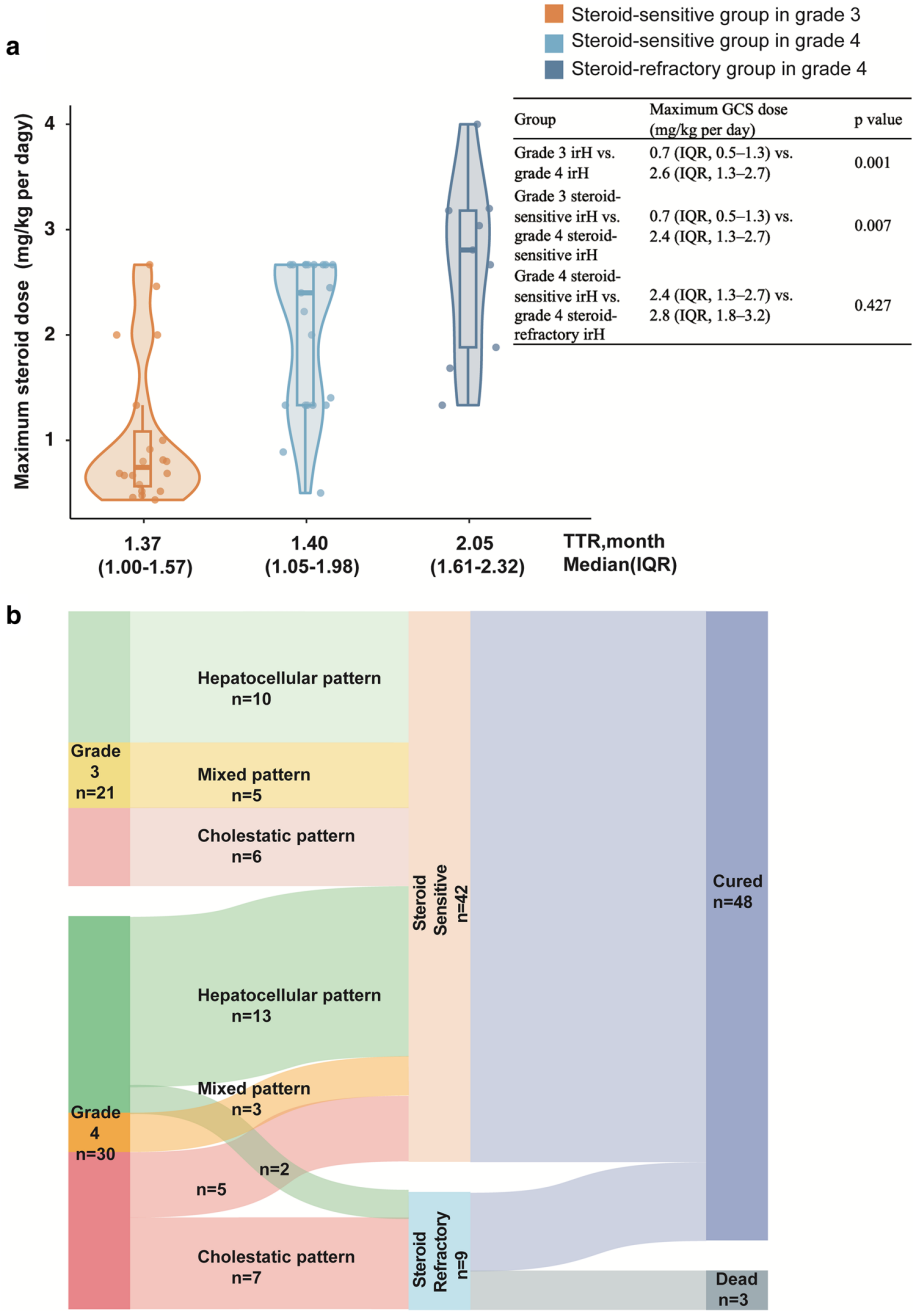
Treatment and outcome of the patients with high-grade immune-related hepatotoxicity (irH). **A** The violin plot shows the time to resolution (TTR) and the maximum glucocorticoid dose applied to the patients in the steroid-sensitive group and steroid-refractory group with grade 3 irH or grade 4 irH, respectively. **B** The Sankey diagram shows the outcomes of the patients with grade 3 and grade 4 irH with different biochemical classifications of liver injury

Immunotherapy was discontinued, and GCS was subsequently administered to all patients. The maximum GCS dose administered and patient outcomes are shown in Fig. 2A and B, respectively. All patients with grade 3 irH were steroid-sensitive and were completely cured afterward. The median maximum dose of GCS (calculated as methylprednisolone equivalent) was 0.7 (IQR, 0.5–1.3) mg/kg/day, and TTR was 1.37 (IQR, 1.00–1.57) months. A higher dose of glucocorticoids (GCS) was administered to grade 4 irH than grade 3 irH (2.6 [IQR, 1.3–2.7] mg/kg/day vs. 0.7 [IQR, 0.5–1.3] mg/kg/day, *p* = 0.001). In patients with grade 4 irH, 21 (70%) patients were steroid-sensitive with a median maximum corticosteroid dose of 2.4 (IQR, 1.3–2.7) mg/kg/day and TTR of 1.40 (IQR, 1.05–1.98) months. Even with a higher GCS dose (median maximum dose: 2.8 [IQR, 1.8–3.2] mg/kg/day), nine (30%) patients still presented resistance to GCS and required second immunosuppression with a TTR of 2.05 (IQR, 1.61–2.32) months. For patients responsive to GCS, those with grade 4 severity required a larger dose of steroids compared to those with grade 3 (*p* = 0.007), but there was no significant difference in the time taken to achieve remission. For patients resistant to GCS, they needed additional immunosuppressants on top of a larger dose of steroids for treatment, and ultimately, they required a longer time to achieve remission compared to patients of the same severity level (*p* = 0.036). The curative effect of GCS therapy on different clinical subtypes of irH showed a great difference. The effective rate of GCS in the hepatocellular pattern and the mixed pattern was 92% (23/25) and 100% (8/8), while that in cholestatic patterns was 44.4% (8/18). The detailed general characteristics and treatment regimens of the steroid-refractory patients are listed in Supplementary Table S3 and Supplementary Table S4.

As salvage therapy for these patients, all patients (seven patients) with cholestatic patterns were administered intravenous immunoglobulin (IVIG), and one patient was also administered tocilizumab. The two patients with a hepatocellular pattern were treated with MMF and tacrolimus, respectively. Five patients (55.6%) improved within 1 week after the administration of the second immunosuppressive agent, and six patients (66.7%) were cured within 3 months (Supplementary Table S4). However, three patients died: Patient 4, with severe systemic symptoms, eventually died of tumor progression and infection 16 weeks after the diagnosis of irH. Patient 6 died of pseudomembranous invasive tracheobronchial aspergillosis during the treatment of irH. Patient 9 did not receive timely GCS after the discovery of grade 4 irH as GCS was administered after 6 weeks and eventually died because of liver failure (histopathology and imaging details are shown in Supplementary Fig. S1), which indicated that delayed management would result in poor prognosis.

### Histological features of the steroid-refractory patients

Six of the nine steroid-refractory patients underwent liver biopsies at the time of diagnosis as steroid-refractory by the authoritative oncologist. The histological features and immunocytochemistry are listed in Table 3.

**Table 3:** The histopathology of the patients with steroid-refractory irH

Patient no.	The histopathology pattern	IS	Inflammation of liver parenchyma and portal area				Bile duct situation	Immunohistochemistry		The distribution of the inflammatory cell
			Focal necrosis, apoptosis, and focal inflammation (0–4)	Portal inflammation (0–4)	Ballooning degeneration	Cholestasis		CD8+/CD4+	CD3+/CD20+	
1	Hepatitic	MMF	2	3	Mild	None	Normal	CD4+ cells dominated	CD3+ cells were sporadically distributed	Concentrated in both hepatic lobules and portal areas
2	Cholangiopathic	IVIG	0	1	Severe	Observed	Bile duct reaction	CD8+ cells dominated^b^	CD3+ cells were scattered in the liver parenchyma^a^; CD20+ cell was not observed	Mainly concentrated in the portal area
3	Cholangiopathic	IVIG	1	1	None	Observed	Bile duct reaction	CD8+ cells dominated	CD3+ cells concentrated around portal; CD20+ cell was not observed	Mainly concentrated in the portal area
4	Cholangiopathic	IVIG	0	1	None	Observed	Bile duct loss	CD8+ cells dominated	CD3+ cells concentrated around portal	Mainly concentrated in the portal area
5	Hepatitic	Tacrolimus	2	3	None	Observed	Normal	CD8+ cells dominated	CD3+ cells were sporadically distributed	Concentrated in both hepatic lobules and portal areas
6	Cholangiopathic	IVIG	1	3	None	Observed	Bile duct reaction was not obvious	CD8+ cells dominated	CD3+ cells were sporadically distributed	Mainly concentrated in the portal area

*irH* immune-related hepatotoxicity, *IS* immunosuppression, *IVIG* intravenous immune globulin, *MMF* mycophenolate

**Fig. 3:**
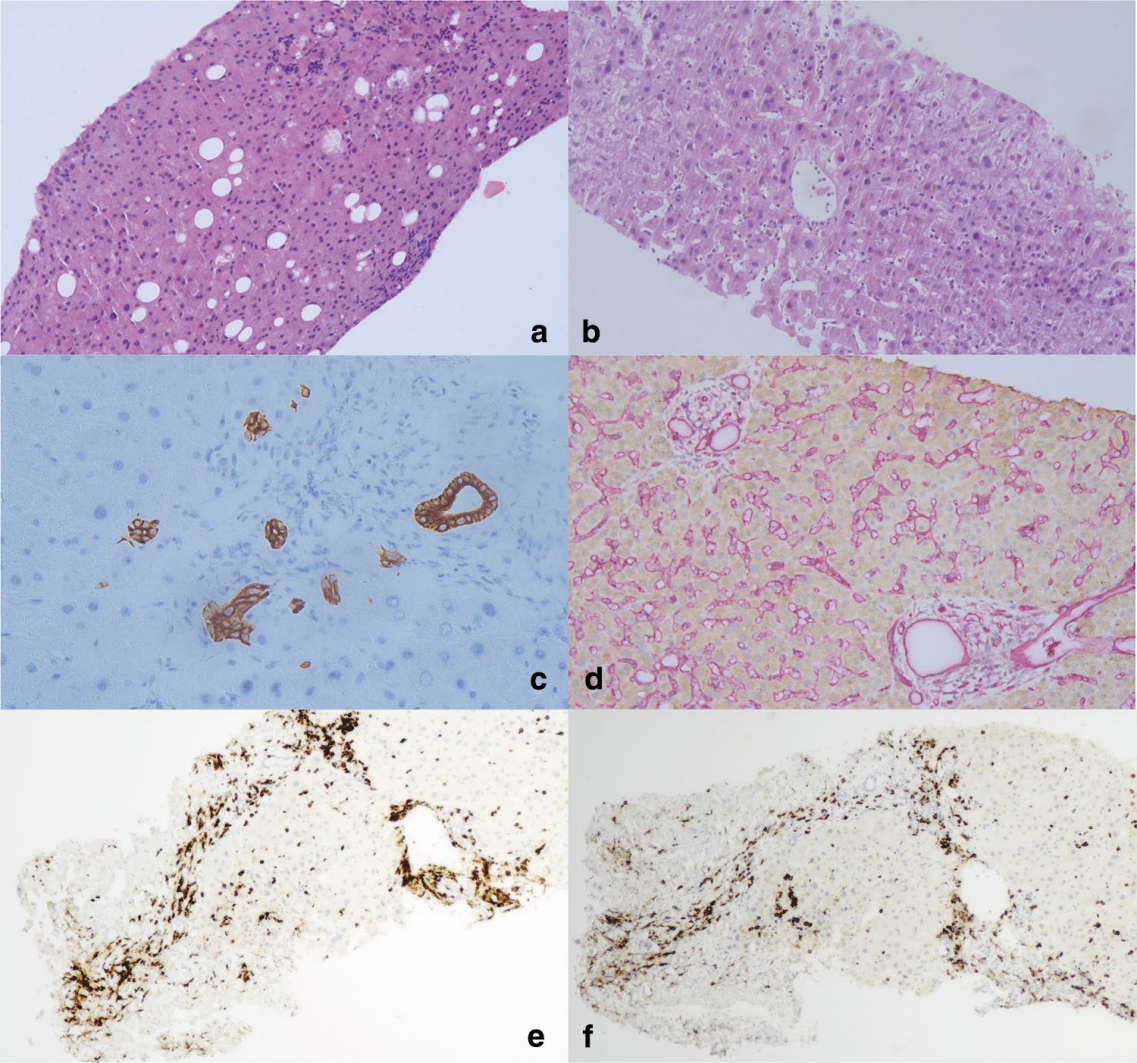
Liver biopsies of steroid-refractory patients. **A** Patient 1: lobular inflammation associated with spotty and focal necrosis with moderate steatosis (hematoxylin–eosin–saffron [HES] ×100). **B**–**C** Patient 2: **B** severe balloon degeneration and cholestasis (HES ×100); **C** bile duct reaction (immunohistochemistry CK19 ×400). **D** Patient 4: the bile duct loss can be observed. Only the vessels appeared in the portal area (CK AE1/AE3&CD34 double-staining ×100). **E**–**F** The immunohistochemistry of the patient 2. **E** CD3+ T cells mainly concentrated around the portal (immunohistochemistry CD3 ×40), **F** CD8 + T cells mainly concentrated around the portal (immunohistochemistry CD3 ×40)

^a^Fig. 3E, the distribution of the CD3+ cell

^b^Fig. 3F, the distribution of the CD8+ cell

The histopathology of Patient 1 and Patient 5 showed a hepatitic pattern. There was no obvious bile duct injury in the portal area, and the main pathological finding was lobular hepatitis, exhibiting spotty necrosis and focal necrosis (Fig. 3A).

The remaining four patients showed a cholangiopathic pattern. Mild inflammatory cells were observed in the hepatic sinusoid and portal area, and the manifestation of lobule injury was mild, with only one patient having severe balloon degeneration (Fig. 3B). The main pathological changes were concentrated in the bile ducts (including bile duct reaction and loss). The bile duct reaction was observed in Patient 2 and Patient 3 (Fig. 3C). In Patient 4, a remarkable bile duct loss was reported (Fig. 3D). There was significant fibrous hyperplasia, small blood vessel hyperplasia, and bile duct reaction, while mild inflammatory cell infiltration in the portal area.

Immunohistochemical results show that in steroid-refractory irH, CD8 + T cells constitute the predominant inflammatory cell type.

### ICPis rechallenge

ICPi was rechallenged in 12 (23.5%) patients (7 patients in the grade 3 irH group and 5 patients in the grade 4 irH group) after recovery from irH. Their characteristics are listed in Supplementary Table S5. Eleven patients were readministered the same ICPi, whereas one patient was changed from PD-1 to PD-L1 inhibitors, and three received concomitant chemotherapy. At the time of the last follow-up, the median rechallenge ICPi dose was 6 (IQR, 3–12), and the median time between severe irH and ICPi retreatment was 26 (IQR, 10–45) weeks. Overall, with a median follow-up time of 9.3 (IQR, 7.5–16.3) months, no irH reoccurred, two patients presented with grade two hypothyroidism, and one presented with grade one eosinophilia and grade two hypothyroidism during the rechallenge. It is worth emphasizing that, compared with patients with mixed or cholestatic patterns, patients with hepatocellular patterns were more likely to resume ICPi treatment after irH (p = 0.035). It may suggest that the type of liver injury may correlate with the probability of retreatment requirement.

## Discussion

To date, this is one of the largest studies reporting patients with high-grade irH and describing their characteristics as well as their management. In our cohort, the incidence rate of irH was 3.49%, and the severe irH rate was 0.96%. The therapeutic regimen we employed indicates that for grade 3 irH, administration of GCS at dosages below the recommended guidelines is sufficient. However, in cases of grade 4 irH, there is a necessity for augmented dosages of GCS. Moreover, we show results of liver biopsies of steroid-refractory patients. In addition, we described the clinical outcomes of rechallenge after high-grade irH and provided hints on the retreatment probability based on the liver injury types.

Given the lack of specific biomarkers, differential diagnosis of irH subtypes has been in the spotlight. Indeed, irH needs a thorough and complete evaluation with a severity classification based on CTCAE, ICPi relevance based on RUCAM, and clinical biochemical classification based on DILI criteria. In our research, the comprehensive evaluation results can be well consistent with the pathological and clinical results, suggesting the rationality of this comprehensive evaluation. There are many clinical subtypes of irH with varied clinical and prognostic characteristics, so multidimensional evaluation was required for irH.

Steroids have been the management backbone for severe irH. Despite that, the guideline recommended the administration of the adequate corticosteroid (1–2 mg/kg/day methylprednisolone equivalents) [9, 10] to limit the toxicity; reducing the dose for high-grade irH was reported to produce a similar rate of response with fewer side effects [15, 16]. In our cohort, in individuals with grade 3 irH, the median maximum dose GCS was lower than that recommended in the guideline, with only one patient experiencing the infection. However, for grade 4 irH, a larger dose of GCS (2–3 mg/kg/day) than what is recommended in the guidelines is still needed, with a treatment course of 6–8 weeks.

Another highlight in our study is the histopathology finding of steroid-refractory irH. For hepatic pattern steroid-refractory irH, pathology shows lymphocyte-dominated inflammation, leading us to primarily use anti-T cell medication in treatment. In contrast, for the cholangiopathic pattern, we observed different histopathological characteristics, including bile duct injury, bile duct reaction, and rare but astonishing bile duct loss. Immunohistochemistry showed lower T-lymphocyte infiltration in these patients compared to those with hepatic pattern, which may partly explain the ineffectiveness of anti-T cell therapy. Previous literature also indicates a lack of efficacy of anti-T cell therapy for this type [11, 12]. Therefore, we hypothesized that besides cellular immunity, other inflammatory mechanisms may damage the bile duct, such as antibody-mediated humoral immunity. A previous study has shown its potential to mediate ICPi-induced hypophysitis and pneumonitis [17]. Another study found lower baseline autoantibody levels, and their changes were associated with organ-specific irAE [18]. IVIG has demonstrated therapeutic efficacy in humoral immune system-mediated autoimmune diseases, both mechanistically and clinically [19]. Considering that IVIG has been applied to other irAEs (pneumonitis, myositis, hematologic irAEs, etc.) [9], and one prior case of steroid-refractory irH with cholangiopathic pattern showed improvement with IVIG [20, 21], we attempted a regimen combining IVIG with GCS to treat seven patients with steroid-refractory irH, thereby proving the efficacy of IVIG in steroid-refractory irH in a cohort for the first time. Bile duct loss reasonably explains the persistent hyperbilirubinemia after adequate GCS administration and the recurrence of irH after IVIG administration for Patient 4. In contrast, patients whose bile ducts were still present had a good prognosis after GCS combined with immunosuppression. So far, a only few cases [12, 22] reported this phenomenon with poor prognosis, for which there is no idea treatment, and the long-term use of glucocorticoids and immunosuppressants is currently the only choice despite the underlying side effects (infection, impact on the tumor response and overall survival, etc.) remaining unclear [23, 24]. Strengthening supportive treatment and controlling opportunistic infection may be more reasonable.

The ICPi rechallenge following high-grade irH is still debatable. Even though permanently discontinuing ICPi is highly recommended by the guideline [9], a recent study on irH retreatment provided encouraging data showing the probability of the rechallenge [25]. In our cohort, seven patients (33.3%) in grade 3 irH and five patients (16.7%) in grade 4 irH were retreated with ICPi after a median time of 26 weeks (IQR, 10–45), and none of them showed irH relapse, although three experienced mild irAEs. This indicates that ICPi rechallenge with a long interval after severe irH, especially grade 4 irH, is feasible and safe. This implication is of great clinical significance, and the described protocol may be of the patient’s best interest, since there are no other treatment options for some patients of these characteristics; thus, retreatment may be a promising choice for them.

Our study had several limitations. It was a retrospective study; therefore, some steroid-refractory patients did not undergo liver biopsy, and some data were missing, such as any previous underlying diseases. The risk factor analysis was constrained by incomplete medication duration data in the non-irH group, focusing only on the types of ICPis, excluding analysis on ICPi sequence or its combination with target therapy or chemotherapy. Yet, our findings are consistent with the previous study, which shows a lower severe irH risk with PD-L1 inhibitor monotherapy or its combination with chemotherapy than with PD-1 inhibitor-based or dual ICPi therapy [26]. Our control group excluded mild irH patients due to difficulties in attributing mild liver abnormalities to ICPis or other treatments and potential oversight of transient mild irH cases. Third, due to the limited number of individuals, IVIG could only be administered to a few steroid-refractory patients. Therefore, future prospective studies are still needed to confirm the reported results.

## Conclusion

Multidimensional evaluation, consisting of clinical and pathological features, is required for patients with irH. Patients who received PD-L1 inhibitors exhibited a lower risk of developing severe irH. For grade 3 irH, a smaller dose of GCS can be administered, while grade 4 irH requires a larger dose than recommended. Pathology may guide the salvage treatment for steroid-refractory irH. Rechallenge of cases with high-grade irH is feasible and safe. However, attention should be paid to a previous biochemical classification of the patients.

## Supplementary Information

Below is the link to the electronic supplementary material.

Supplementary file1 (DOCX 5315 KB)

## Data Availability

All data relevant to the study are included in the article or uploaded as supplementary information.
