# Supplementary material for: Management and treatment of severe immune-related hepatotoxicity based on clinical and pathological characteristics
Source: Hepatol Int. 2024 Jul 2;18(6):1770–80. doi: 10.1007/s12072-024-10688-0 (PMC11632075; doi:10.1007/s12072-024-10688-0)
Supplement: Supplementary file 1 — Supplementary file1 (DOCX 5315 KB) [file 12072_2024_10688_MOESM1_ESM.docx]

**Supplementary Materials for**

Management and Treatment of Severe Immune-related Hepatotoxicity Based on Clinical and Pathological Characteristics

Supplementary methods

**Laboratory parameters which were recorded for the severe irH group**

Laboratory parameters consisted of hematology, alanine aminotransferase (ALT; normal range, 9–50 IU/L), aspartate aminotransferase (AST; normal range, 15–40 IU/L), gamma-glutamyl transferase (GGT; normal range, 10–60 IU/L), alkaline phosphatase (ALP; normal range, 45–125 IU/L), total bilirubin (TBil; normal range, 5.1–22.2 μmol/L), direct bilirubin (Dbil; normal range, 0–6.8 μmol/L), autoimmune serology (total serum immunoglobulin G, anti-nuclear antibody [ANA], anti-smooth muscle antibody, type 1 anti-liver kidney microsomal antibody, or soluble liver/liver–pancreas antibody), and viral hepatitis serology (hepatitis A, B, C, and E virus [HAV, HBV, HCV, and HEV]).

**Propensity-score matching**

A logistic regression model was fitted, including ten baseline covariates: age, sex, tumor type, tumor stage, diabetes, hypertension, prior myocardial infarction, coronary artery disease, heart failure, and cerebrovascular disease. The manifestation status of severe irH, categorized as either the absence of irH or the presence of severe irH, was entered into the model as the dependent variable. Matching was performed using a 1:1 nearest-neighbor matching protocol without replacement, with a match tolerance of 0.02.

Supplementary Tables

**Supplementary Table S1** Univariate and multivariate conditional logistic analysis of risk factors for severe irH

| Variates | Univariate analysis | Multivariate analysis | |
| --- | --- | --- | --- |
|  | P value | OR (95% CI) | P value |
| HBV infection, yes vs. no | **0.0544** | 3.844 (0.73595-20.0796) | 0.1104 |
| HCV infection, yes vs. no | 0.571 |  |  |
| Fatty liver disease, yes vs. no | 0.999 |  |  |
| Autoimmune diseases, yes vs. no | 0.998 |  |  |
| Alcohol intake, yes vs. no | **0.0674** | 2.685 (0.63776-11.3056) | 0.1781 |
| Liver metastasis, yes vs. no | 0.782 |  |  |
| Anti-PD-1, yes vs. no | **0.0674** | 1.797 (0.42607-7.5828) | 0.4247 |
| Anti-PD-L1, yes vs. no | **0.0078** | **0.230 (0.05899-0.8969)** | **0.0343** |
| Anti-CTLA-4+anti-PD-1/PD-L1, yes vs. no | 0.998 |  |  |
| Target therapy alone, yes vs. no | 1 |  |  |
| Chemotherapy alone, yes vs. no | 0.416 |  |  |
| Target therapy plus chemotherapy, yes vs. no | 0.206 |  |  |

Abbreviations: CTLA-4, cytotoxic T-lymphocyte antigen-4; CI, confidence interval; HBV, hepatitis B virus; HCV, hepatitis C virus; irH, immune-related hepatotoxicity; OR, Odds Ratio; PD-1, programmed death protein-1; PD-L1, programmed death ligand-1.

**Supplementary Table S2** Details of anti-tumor treatment for severe irH patients

| **Patient** | **irH grade** | **Tumor type** | **Stage** | **ICPi** | **Number of ICPi Cycle** | **Biochemical classification of liver injury** |
| --- | --- | --- | --- | --- | --- | --- |
| 001 | 3 | Cholangiocarcinoma | IV | Toripalimab | 5 | Cholestatic |
| 002 | 4 | NSCLC | Ⅳ | Pembrolizumab | 2 | Hepatocellular |
| 003 | 3 | Endometrial cancer | Ⅲc | Tislelizumab | 6 | Cholestatic |
| 004 | 4 | NSCLC | IV | Camrelizumab | 1 | Cholestatic |
| 005 | 3 | Renal clear cell carcinoma | IV | Camrelizumab | 3 | Mixed |
| 006 | 3 | Renal clear cell carcinoma | IIIb | Nivolumab+  Ipilimumab | 2 | Hepatocellular |
| 007 | 4 | Adrenocortical Carcinoma | IV | Pembrolizumab | 1 | Cholestatic |
| 008 | 4 | NSCLC | IV | Pembrolizumab | 8 | Hepatocellular |
| 009 | 3 | NSCLC | IIIa | Pembrolizumab | 1 | Hepatocellular |
| 010 | 4 | NSCLC | IIIb | Camrelizumab | 1 | Hepatocellular |
| 011 | 4 | NSCLC | IIIa | Pembrolizumab | 1 | Cholestatic |
| 012 | 3 | Thymic squamous cell carcinoma | IV | Pembrolizumab | 1 | Hepatocellular |
| 013 | 4 | HCC | IIIb | Sintilimab | 1 | Hepatocellular |
| 014 | 4 | Thymoma | IV | Pembrolizumab | 2 | Cholestatic |
| 015 | 3 | Ovarian cancer | IV | Pembrolizumab | 4 | Mixed |
| 016 | 3 | NSCLC | IV | Sintilimab | 2 | Hepatocellular |
| 017 | 3 | NSCLC | IV | Pembrolizumab | 1 | Mixed |
| 018 | 4 | NSCLC | IV | Pembrolizumab | 4 | Cholestatic |
| 019 | 4 | SCLC | IV | Camrelizumab | 4 | Hepatocellular |
| 020 | 3 | Hypopharyngeal cancer | IIIb | Camrelizumab | 1 | Hepatocellular |
| 021 | 4 | HCC | IV | Tislelizumab | 1 | Hepatocellular |
| 022 | 4 | NSCLC | IV | Pembrolizumab | 2 | Cholestatic |
| 023 | 3 | Endometrial cancer | IIIa | Pembrolizumab | 1 | Hepatocellular |
| 024 | 3 | NSCLC | IV | Pembrolizumab | 2 | Cholestatic |
| 025 | 3 | Stomach cancer | IV | Sintilimab | 2 | Hepatocellular |
| 026 | 3 | Rectal cancer | IV | Durvalumab | 1 | Cholestatic |
| 027 | 3 | Mesothelioma | IV | Nivolumab+  Ipilimumab | 3 | Hepatocellular |
| 028 | 4 | Stomach cancer | IIIa | Nivolumab | 4 | Cholestatic |
| 029 | 3 | Esophageal cancer | IV | Camrelizumab | 1 | Mixed |
| 030 | 3 | HCC | IIIb | CS1003 | 11 | Cholestatic |
| 031 | 4 | Rectal cancer | IV | Pembrolizumab | 2 | Hepatocellular |
| 032 | 4 | SCLC | IV | Tislelizumab | 1 | Hepatocellular |
| 033 | 4 | ICC | IV | Durvalumab | 5 | Cholestatic |
| 034 | 4 | HCC | IV | Durvalumab | 3 | Cholestatic |
| 035 | 4 | HCC | IV | Tislelizumab | 11 | Hepatocellular |
| 036 | 4 | NSCLC | IV | Pembrolizumab | 4 | Hepatocellular |

**Supplementary Table S2 (continued)**

| **Patient** | **irH grade** | **Tumor type** | **Stage** | **ICPi** | **Number of ICPi Cycle** | **Biochemical classification of liver injury** |
| --- | --- | --- | --- | --- | --- | --- |
| 037 | 3 | Esophageal cancer | IV | Pembrolizumab | 1 | Hepatocellular |
| 038 | 3 | Rectal cancer | IV | Tislelizumab | 2 | Mixed |
| 039 | 4 | NSCLC | IV | Pembrolizumab | 1 | Mixed |
| 040 | 4 | Rectal Cancer | IIIa | Pembrolizumab | 4 | Hepatocellular |
| 041 | 4 | Thymic squamous cell carcinoma | IV | Tislelizumab | 2 | Cholestatic |
| 042 | 3 | NSCLC | IV | Toripalimab | 2 | Hepatocellular |
| 043 | 4 | NSCLC | IV | Tislelizumab | 2 | Cholestatic |
| 044 | 4 | Renal clear cell carcinoma | IIIb | Toripalimab | 2 | Hepatocellular |
| 045 | 4 | NSCLC | IIIb | Pembrolizumab | 3 | Hepatocellular |
| 046 | 4 | Stomach cancer | IIIb | Tislelizumab | 2 | Cholestatic |
| 047 | 3 | HCC | IIIb | Tislelizumab | 2 | Cholestatic |
| 048 | 4 | NSCLC | IIIb | Tislelizumab | 1 | Hepatocellular |
| 049 | 4 | Endometrial cancer | IV | Tislelizumab | 3 | Hepatocellular |
| 050 | 4 | NSCLC | IV | Serplulimab | 2 | Mixed |
| 051 | 4 | NSCLC | IV | Pembrolizumab | 5 | Mixed |

Abbreviations: HCC, hepatocellular carcinoma; ICC, intrahepatic cholangiocarcinoma; NSCLC, non-small-cell lung cancer; SCLC, small-cell lung cancer.

**Supplementary Table S3** The general characteristics of the patients with steroid-refractory irH

| **Patient** | **ICPi related to irH** | **Number of ICPi Cycle** | **TTO, days** | **Biochemical classification** | **Clinical symptoms** | **Serum autoantibody** | **Therapy for irH (max dose of steroid/IS)** | **Outcomes** |
| --- | --- | --- | --- | --- | --- | --- | --- | --- |
|  |  |  |  |  |  |  |  |  |
| 1 | Camrelizumab | 4 | 84 | Hepatocellular | Anorexia | ANA (+): 1:80 | MP 80mg/MMF | Cured |
| 2 | Pembrolizumab | 1 | 6 | Cholestatic | Jaundice, fever | ANA (-) | MP 160mg/IVIG | Cured |
| 3 | Pembrolizumab | 4 | 76 | Cholestatic | Anorexia, jaundice | ANA (+): 1:80 | MP 160mg/IVIG | Cured |
| 4 | Pembrolizumab | 2 | 42 | Cholestatic | Vomit, anorexia, jaundice, fever, joint pain | ANA (+): 1:80 | MP 160mg/IVIG | Dead |
| 5 | Pembrolizumab | 4 | 95 | Hepatocellular | Jaundice, fever | ANA (-) | MP 160mg/ Tacrolimus | Cured |
| 6 | Tislelizumab | 2 | 61 | Cholestatic | Vomit, anorexia, jaundice, fever | ANA (-) | MP 240mg/IVIG | Dead |
| 7 | Pembrolizumab | 1 | 12 | Cholestatic | Vomit, anorexia, jaundice | ANA (+): 1:80 | MP 160mg/IVIG | Cured |
| 8 | Nivolumab | 5 | 117 | Cholestatic | Anorexia, jaundice, fever | ANA (-) | MP 160mg/IVIG | Dead |
| 9 | Pembrolizumab | 2 | 34 | Cholestatic | Anorexia, jaundice, fever, joint pain | ANA (+): 1:100 | MP 160mg/IVIG, Tocilizumab | Cured |

Abbreviations: irH, immune-related hepatotoxicity; ICPi, immune checkpoint inhibitor; TTO, time to onset; IS, immunosuppression; MP, methylprednisolone; IVIG, intravenous immune globulin; MMF, mycophenolate.

**Supplementary Table S4** The treatment and outcomes of the patients with steroid-refractory irH

| **Patient** | **Second IS (Day 1)** | **Days from admit to second IS** | **irAE grade at admit** | **Day 1 irAE grade** | **Day 2 irAE grade** | **Day 3 irAE grade** | **Day 4 irAE grade** | **Day 5 irAE grade** | **Day 6 irAE grade** | **Day 7 irAE grade** | **Day 1 steroid given** | **Day 2 steroid given** | **Day 3 steroid given** | **Day 4 steroid given** | **Day 5 steroid given** | **Day 6 steroid given** | **Day 7 steroid given** | **Improved in 1 week?** | **Improved in 90 days?** | **Recurrence** |
| --- | --- | --- | --- | --- | --- | --- | --- | --- | --- | --- | --- | --- | --- | --- | --- | --- | --- | --- | --- | --- |
| 1 | MMF | 9 | 3 | 4 | 4 | 4 | 3 | 3 | 3 | 3 | MP60 | MP60 | MP40 | MP40 | MP40 | MP40 | MP40 | YES | YES | NO |
| 2 | IVIG | 11 | 4 | 4 | 4 | 4 | 3 | 3 | 3 | 3 | MP160 | MP160 | MP 80 | MP 80 | MP80 | MP80 | MP80 | YES | YES | NO |
| 3 | IVIG | 22 | 4 | 4 | 3 | 3 | 3 | 3 | 3 | 3 | MP160 | MP160 | MP160 | MP160 | MP 80 | MP80 | MP80 | YES | YES | NO |
| 4 | IVIG | 4 | 4 | 4 | 3 | 3 | 3 | 2 | 2 | 2 | MP160 | MP160 | MP160 | MP160 | MP160 | MP80 | MP80 | YES | NO | Not applicable |
| 5 | Tacrolimus | 7 | 4 | 4 | 4 | 3 | 3 | 3 | 3 | 3 | MP160 | MP160 | MP160 | MP160 | MP160 | MP120 | MP120 | YES | YES | NO |
| 6 | IVIG | 4 | 4 | 4 | 4 | 4 | 4 | 4 | 4 | 4 | MP240 | MP240 | MP160 | MP160 | MP160 | MP160 | MP160 | NO | NO | Not applicable |
| 7 | IVIG | 20 | 4 | 4 | 3 | 3 | 3 | 3 | 3 | 3 | MP160 | MP160 | MP160 | MP80 | MP80 | MP80 | MP80 | NO | YES | NO |
| 8 | IVIG | 7 | 4 | 4 | 4 | 4 | 3 | 3 | 3 | 4 | MP160 | MP160 | MP160 | MP160 | MP80 | MP80 | MP160 | NO | YES | NO |
| 9 | IVIG plus Tocilizumab | 49 | 4 | 4 | 4 | 4 | 4 | 4 | 4 | 4 | MP80 | MP80 | MP80 | MP40 | MP40 | MP40 | MP40 | NO | NO | Not applicable |

Abbreviations: irH, immune-related hepatotoxicity; IS, immunosuppression; MP, methylprednisolone; IVIG, intravenous immune globulin; MMF, mycophenolate; irAE, immune-related adverse events.

**Supplementary Table S5** The characteristics of patients with ICPi rechallenge

| **Variables** | **Rechallenge（N=12）** |
| --- | --- |
| Age (years, median, IQR) | 60 (53-65) |
| Male, n (%) | 9 (75) |
| HBV infection, n (%) | 2 (16.7) |
| Alcohol Use, n (%) | 2 (16.7) |
| Biochemical classification of liver injury^*^, n (%) |  |
| Hepatocellular | 9 (75) |
| Mixed | 1 (8.3) |
| Cholestatic | 2 (16.7) |
| ICPi Categories, n (%) |  |
| Anti-PD-1 | 9 (75) |
| Anti-PD-L1 | 1 (8.3) |
| Anti-CTLA-4+anti-PD-1 | 2 (16.7) |
| Number of rechallenge ICPi cycle (median, IQR) | 6 (3-12) |
| The duration between irH and rechallenge, weeks (median, IQR) | 26 (IQR,10-45) |
| Accompanying irAE, n (%) |  |
| Hypothyroidism | 3 (25) |
| Eosinophilia | 1 (8.3) |

Abbreviations: HBV, Hepatitis B Virus; ICPi, Immune checkpoint inhibitor; irH, immune-related hepatotoxicity; irAE, immune-related adverse event; IQR, Interquartile range.

* Comparison was performed among patients with hepatocellular pattern versus mixed/cholestatic pattern, the p-value= 0.035 (Fisher's exact test).

Supplementary Figures


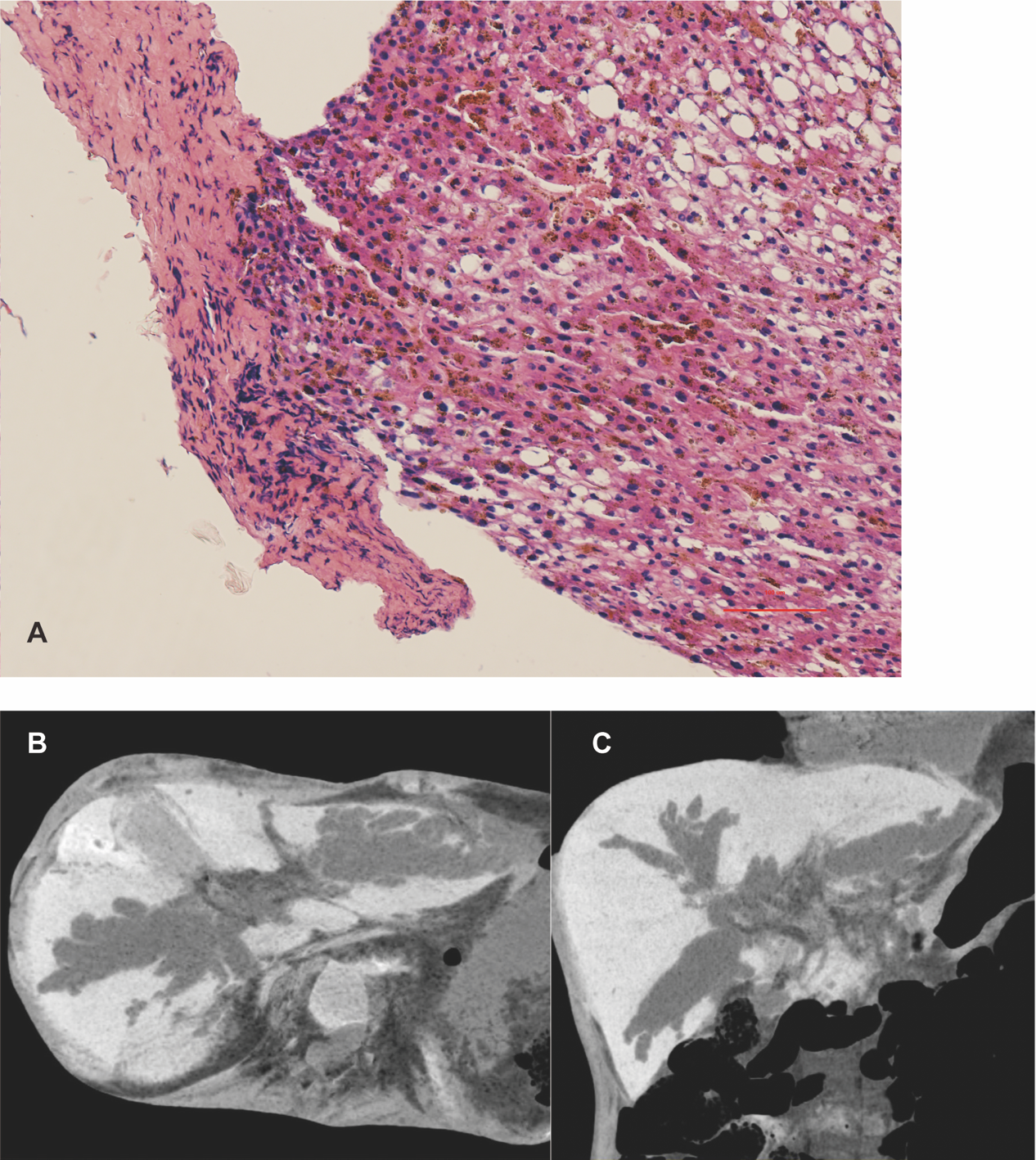


**Supplementary Figure S1 The histopathology and imaging of the patient 7**

(**A**) The liver biopsy of the patient 7 when 4 weeks after the diagnosis of irH. The hepatocytes are steatosis with ballooning change, large areas of collagen and cholestasis are seen. (Hematoxylin-eosin-saffron (HES) x150)

(**B-C**) The abdominal magnetic resonance imaging (**B**) and computerized tomography (**C**) of the patient 7. The wall of the common hepatic duct, common bile duct, and gallbladder was thickened with enhancement, and inflammatory changes were possible. The lumen of the extrahepatic bile duct was not dilated.
